# Mutual-Aided INS/Vision Navigation System Analysis and Optimization Using Sequential Filtering with State Recalculation

**DOI:** 10.3390/s23010079

**Published:** 2022-12-22

**Authors:** Ayham Shahoud, Dmitriy Shashev, Stanislav Shidlovskiy

**Affiliations:** Faculty of Innovative Technology, Tomsk State University, 36 Lenin Ave, 634050 Tomsk, Russia

**Keywords:** integrated navigation, asynchronized measurements, sequential filtering, clustering, delay, filter recalculation, mutual aiding, IMU, GPS, Kd-Tree

## Abstract

This paper presents the implementation of a mutual-aided navigation system for an aerial vehicle. Employing all available sensors in navigation is effective at maintaining continuous and optimal results. The images offer a lot of information about the surrounding environment, but image processing is time-consuming and causes timing problems. While traditional fusion algorithms tend to reduce the delay errors or ignore them, this research depends on state estimation recalculation during the delay time and on sequential filtering. To reduce the image matching time, the map is processed offline, then key point clusters are stored to avoid feature recalculation online. The sensors’ information is used to bound the search space for the matched features on the map, then they are reprojected on the captured images to exclude the unuseful part from processing. The suggested mutual-aided form compensates for the inertial system drift, which enhances the system’s accuracy and independence. The system was tested using data collected from a real flight using a DJI drone. The measurements from an inertial measurement unit (IMU), camera, barometer, and magnetometer were fused using a sequential Kalman Filter. The final results prove the efficiency of the suggested system to navigate with high independency, with an RMS position error of less than 3.5 m.

## 1. Introduction

Using all of the available information in the navigation process is one of the main characteristics of any modern automated system, both in ground and aerial applications. The most accurate is the navigation system, but a higher performance can be obtained. Nowadays, it is not common to see a navigation system that relies only on a single sensor. Fusing measurements from more than one sensor to obtain a more robust and optimal navigation solution has been widely adopted. In navigation applications, the employment of the sensors’ information in their real temporal and spatial (geolocation) context is a key point for high performance and reliability. The importance of such employment is also obvious in the case of the necessity of using the calculated carrier position (the aerial vehicle position) in localizing other existing objects (key points, structure, person, or other special areas) in the navigation environments. Such applications exist in remote sensing, and mapping, for example, simultaneous localization and mapping (SLAM), image registration, building digital surface models (DSM), search and rescue, and surveillance. Computer vision-based navigation systems are an essential part of the previous applications because mostly optical sensors, such as cameras, are used to execute the missions [1,2]. 

Sensors’ asynchronization and image-processing time delays lead to errors in the carrier position, and hence, in the desired task. After capturing an image, it undergoes a time-costly processing procedure to extract useful position information from it. In the case of not compensating for the image-processing time delay, the resulting map will be corrupted by errors, and automated search and rescue missions (or intelligent search and rescue missions) might be dangerous instead of being helpful. In general, any information that will be extracted from the image concerning the geolocation will be corrupted by errors. Furthermore, the vehicle path control will not be accurate. This research focuses on solving the problems of image-processing time delay by decreasing the processing time and employing the image information at “the accurate time” that corresponds to “the actual position”, which is at the “capturing moment”. The aforementioned process has obvious direct impacts on a lot of vision-based navigation tasks (by means of improving the accuracy and making the implementation easier), such as surveillance, path tracking, and mapping in the case of depending on the carrier position in these missions.

The common sensors that are widely used in navigation are inertial sensors, GPS, cameras, barometers, magnetometers, radars, and sonars. Most of the previous sensors are available nowadays in small sizes, low prices, light weight, and with well-supported platforms (software and hardware). The GPS gives accurate and absolute measurements, but it suffers from outages and multipath problems. Inertial navigation system (INS) sensors are totally independent, but they suffer from drift over time because of the mathematical integration of the sensors’ output errors. The aforementioned problems motivate researchers in the navigation domain to exclude GPS or replace it with more independent systems. Such systems could be computer vision-based navigation systems, for example [3].

One of the famous fusion algorithms that are used in navigation applications is the Kalman filter (KF), which depends on the state space representation. The Kalman filter gives optimal solutions in the case of Gaussian noise and linear systems. Since such conditions are not always available, the extended Kalman filter (EKF) and unscented Kalman filter offer good alternatives to reduce the nonlinearity and the color noise problems, respectively. In addition, particle filtering-based navigation works well in the case of very complex systems, but it is a time-consuming process. In the case of multi-sensor implementation, in addition to noise and sensor mismodeling, other problems arise, such as synchronization between the sensors and measurement delay. All the previous problems are because of the fact that in the real world such ideal sensors do not exist. A sequential KF offers a good option in the case of integrating sensors with different models, working rates, and the required processing times. Sequential filtering refers to correcting or updating the system state each time an individual measurement from any sensor arrives [3,4].

**Figure 1:**
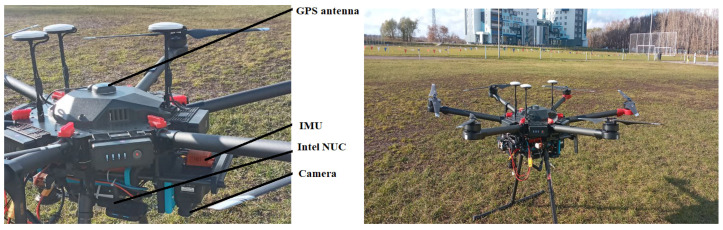
The DJI Matrice Pro 600 at the launch point is shown in the right image. The attached equipment on the drone is shown in the left image.

Images offer a lot of information that could be beneficial in localization, such as objects’ sizes, distances to other objects, objects’ distribution in the environment (such as topological maps), and the environment’s texture. The image-processing and artificial intelligence (AI) techniques are basic tools to implement such intelligent and robust visual navigation systems. The visual navigation calculated position could be absolute, such as scene matching, or incremental, such as visual odometry [5]. In this work, the fusion between multiple navigational sensors is adopted, and mutual aiding between the INS and the vision-based system is realized. The optimization of the image-processing time delay and accurate output continuity are obtained by several procedures. Firstly, while scene matching with a reference map is carried out, the reference images are processed offline, and feature clusters are created from each image and stored in a Kd-Tree to avoid recalculating them online, which leads to a speed-up of the searching process. Secondly, a recalculation of the EKF using state information stored during the image-processing time (from the capturing moment until the visual measurement is ready) is carried, which reduces the errors caused by the time delay problem. Thirdly, while navigation based on scene matching is used to compensate for the INS drift, the integrated navigation solution is used to limit the search area on the reference image. At the same time, the clusters’ centers on the reference images are reprojected on the captured image to bound the search space on it also. Finally, sequential filtering is adopted to fuse any available measurements in the process, which solves the problem of multi-rate multi-sensors. The aforementioned steps make up the main contributions of this paper, beginning from map feature clustering, map features stored in the Kd-Tree, captured image cropping according to the INS measurements, and finally, the recalculation of the sequential KF to solve the image-processing time delay. All the previous steps help to optimize the results and maintain measurement continuity. The final system was tested using data collected with a DJI Matrice 600 Pro equipped with cameras, an IMU, and all needed sensors. The drone is shown in Figure 1. During the experiments, GPS data (velocity and position) were also collected to be used as reference data for the results of the proposed algorithm. The GPS data were not included in the navigation calculations, i.e., after the data collection (images, IMU data, barometer data, and compass data) and applying the algorithm to them, the results were drawn along with the reference GPS measurements. Actually, excluding the GPS from the integration was due to the fact that the GPS is controlled by a provider, so depending on it will affect the system’s overall independence. Furthermore, scene matching was adopted to replace the GPS since it is considered more independent and it also provides the absolute position of the drone (which is needed to compensate for INS drift).

The rest of this paper is organized as follows. Section 2 presents related studies, Section 3 presents mutual aiding and sequential filtering, Section 4 presents reducing the search space in the feature-matching process, Section 5 presents the system state dynamics and integrated navigation algorithm, Section 6 presents the implementation and realization, Section 7 presents the analysis of the results, and Section 8 presents the conclusion.

## 2. Related Study

Employing all available information to obtain an optimal and robust navigation solution is a matter of interest for studies nowadays. Fusion can be performed at the level of navigation systems, called “loose coupling”, or at the level of the sensors’ raw data, called “tight coupling”. The mutual error compensation at the level of the sensors or at the subsystems level helps to improve the final results, which is called mutual aiding [6]. In [7], an image-aided inertial navigation system was presented for an octocopter. A tightly coupled integration between inertial data and the visual navigation system to overcome GPS outages was realized. In [8], they introduced the design of an optimal estimation algorithm for the multi-sensor fusion of a redundant MEMS gyro system. Multi-sensor fusion and integration: approaches, applications, and future research directions were presented in [9]. In [10], they explained the effects of delay caused by real-time image acquisition and feature tracking in a previously documented vision-augmented inertial navigation system. In [11], they presented time-delay systems: modeling, analysis, estimation, control, and synchronization. In [12], they introduced a visual compass for a downward-looking camera for an unmanned aerial vehicle (UAV) based on key point clustering. In [13], they presented false/true and matching classifications using a convolutional neural network (CNN) for a scene-matching-based navigation system. In [14], a robust sequential Kalman filtering algorithm based on Huber was presented. The algorithm combines sequential Kalman and Huber filtering. In [15], they introduced an adaptive sequential Monte Carlo filter for indoor positioning and tracking with Bluetooth low-energy beacons.

Some studies have solved the delay problems through hardware improvements, such as depending on high-performance CPUs, fast communication protocols, and expensive sensors. These solutions increase the cost and might not be suitable when limited onboard computers are needed. Using redundant sensors to obtain the optimal solutions also increases the cost and makes the synchronization problem harder as the number of sensors increases. Furthermore, in an integrated system, extensively focusing on individual sub-systems’ specifications (noise, output types), and assuming that integration will solve all problems and outcomes, is a trap into which some researchers might fall.

In this work, mutual aiding reduced the time processing for the visual system and enhanced the INS accuracy. After projecting the INS position on the map, the reprojection process on the captured images then reduced the matching process time. Furthermore, sequential filtering with the EKF recalculation overcame the image-processing time error, even if it consumed a little bit more time at each recalculation step. Applying the suggested methods to real data collected from a DJI drone is an important step for an online working mode in the future.

## 3. Mutual Aiding and Sequential Filtering

Integrated navigation systems are realized in different forms according to the integration purposes and the sub-system or sensor specifications. The systems that care about improving the accuracy of the final results usually depend on fusion algorithms that obtain some optimality criteria, such as KF. KF works well in the case of a linear system derived from Gaussian noise, which is not always the real situation. The EKF solves the nonlinearity problem by linearizing the state around a certain work point. Increasing the robustness of the overall system could be established by using redundant sensors, which work in parallel or in configurations that make the system more robust against variations in power, amplitudes, shocks, and environmental conditions. Integrating the sensors’ or systems’ final results in parallel is enough to add some reliability to the overall system, but sometimes the opposite is true or more convenient. For example, INS/GPS integration, in its loose form, requires four GPS satellites to be in view, which is not always possible, while in a tight configuration form, the INS measurements are fused with the raw data from the GPS (pseudo-range and doppler) even for one satellite. As a result, the INS drift is corrected using only a raw measurement, the GPS clock bias is estimated, and the overall system robustness against weather and an environment with weak GPS signals will increase [3,15].

This work presents an integrated navigation system that uses all of the available information from all sensors, i.e., the IMU, compass, barometer, and camera. The objectives of the fusion were to improve both the independency and the accuracy of the integrated system. All sensors were corrupted with noise, which was considered to be Gaussian. The nonlinearity was solved by using the EKF. Each sensor or system had a working frequency, and each one had a processing time after being digitalized. To solve the multi-rate problem, sequential filtering was adopted, i.e., the system was updated with the available measurements when they were ready. A recalculation during the image-processing period took place once the visual measurement was ready from the capturing moment [10].

**Figure 2:**
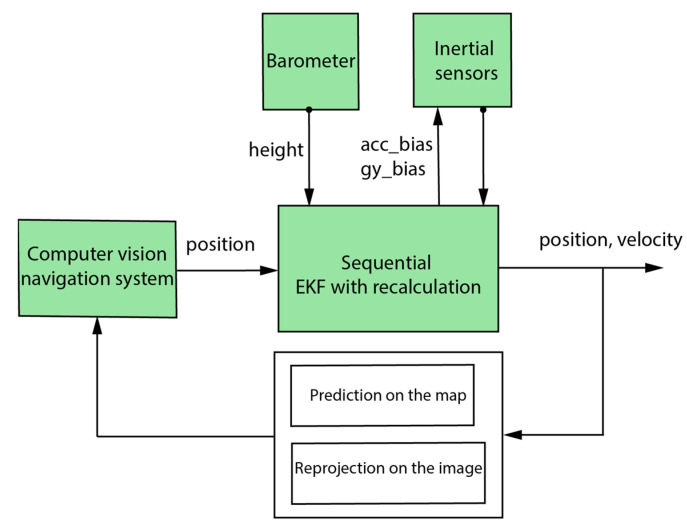
General diagram of the mutual aiding-based navigation system.

Scene matching based on local features was used in the position calculation, where the image processing took a long time. To reduce the matching time, a mutual coupling form was adopted between the INS and the visual navigation system. The actual position of the drone was used to bound the searching area for matched features on the map. At the same time, the INS information, in addition to the map features, was used to bound the searching area on the captured image. In addition, to overcome the image-processing time errors, a recalculation took place each time a vision output was ready from the image capture moment, which represented the real drone position at the capturing moment. The system diagram is shown in Figure 2.

### Delay Analysis and EKF Recalculation 

The sensors involved in the integrated systems do not follow the same clock, and they also might have different working rates. Furthermore, each sensor needs its special processing, filtering, and scaling. As a result, problems related to time delay and asynchronization will appear. Such errors affect the accuracy and even the stability of the system. A perfect solution for such problems might not exist. Sometimes it is possible to ignore these errors when they are small, according to the application, or by using higher-performance CPUs or sensor versions. Additionally, optimizing the algorithms or relying on parallel programming could be an effective solution. In the case of fixed delay, the constant compensation model might be enough. In image-processing applications, the worst-case time delay is large and variable with time according to the CPU processing load [6,15].

**Figure 3:**
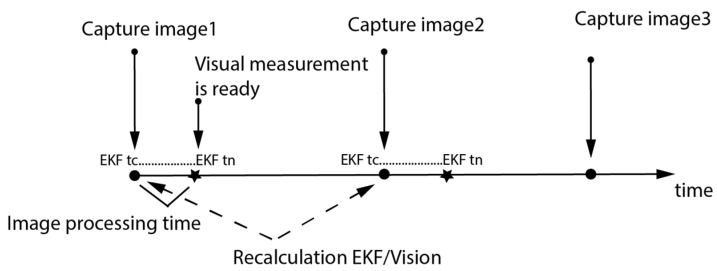
Recalculation using EKF. When a vision measurement was ready, the EKF was recalculated from the capturing moment to the actual moment (t_n_-t_c_) after updating the state with visual measurements at “t_c_”. Otherwise, the EKF was carried out between the INS and the barometer only.

In this study, the feature detection and description procedure were time-varying according to the number of detected features in each image, and they contributed to the largest part of the visual navigation algorithm computation load. Knowing the image-capturing moment “t_c_“ and the image-processing time, this work suggested recalculating the EKF to update the state using the visual measurements in the accurate moments. Sequential filtering with KF by updating the state process using any measurement when it was ready was a good KF choice to realize the suggested recalculation. The vision-based navigation system results were used to update the EKF state, while projecting the INS results to the map reduced the matching process because matching took place with only some map clusters. Sequential recalculation using an EKF navigation chart is illustrated in Figure 3.

The EKF continued normally between the barometer and the INS until the moment when the visual measurement was ready. Then, the vision update took place at the capturing moment, and the EKF was recalculated from that moment to the moment when the vision results were ready. The image-capturing moment corresponded to the drone position, which was calculated from that image. The other sensors’ responses and data processing times were negligible compared to the image-processing time.

## 4. Reducing the Search Space in the Feature-Matching Process

Image matching could be realized either based on extreme correlation techniques, such as cross-correlation, or using feature-matching techniques [16]. Correlation-based techniques require image alignment, i.e., other sensors are needed to rotate and scale both images to have the same scale and orientation. The feature-based matching algorithms, which employ local features such as scale-invariant feature transform (SIFT), speeded-up robust features (SURF), and ORB, are robust to variations in scale and orientation. It is worth mentioning that it is enough to have some corresponding features (four points, for example, in the perspective-n-point (PnP) algorithm) to calculate the transformation (rotation and translation caused by camera movement) between two images, i.e., only a part of the image is needed, so losing a part of the image or cropping it will not cancel the matching process [17]. Feature-based matching is time-consuming in detection and description, while matching can be established using Cartesian distance. 

Several steps were adopted in this work to enhance the matching process. In the first step, since the flight map was already prepared offline, processing the reference map was also performed offline, so features were clustered and stored with no need to recalculate them online. In the second step, the INS calculation was used to bound the search area for features using the geographical coordinates of the drone calculated by the INS to limit the targeted clusters on the reference map. Furthermore, the selected clusters’ centers on the map were projected on the captured image plane and then used to crop the useful part of the captured image. The previous steps were carried out using the orientation and the camera’s internal parameters, such as the field of view (FOV) and focal length. The local level coordinate center, the INS center of mass, and the camera coordinates were assumed to be identical.

**Figure 4:**
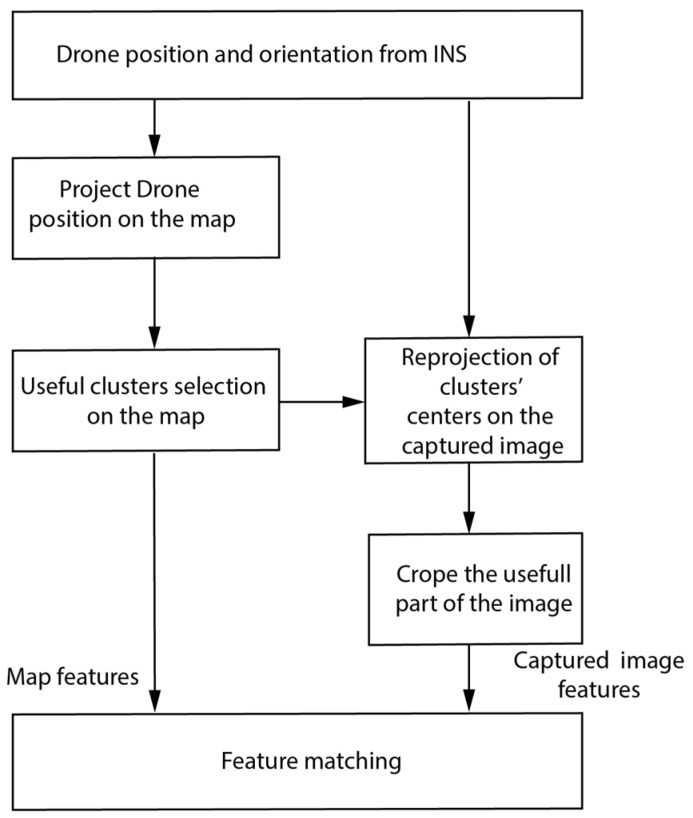
The diagram for features-matching enhancement using the drone INS measurements.

The reference map was processed offline, i.e., image enhancement, feature detection, and a description on a 64-byte vector were performed using SURF. Calculating the transformation between two captured images was performed using a 3D–2D matching algorithm (PnP was adopted). PnP requires four points (corresponding features) only to calculate a unique solution, and any additional points are used in the optimization. PnP was applied along with random sample consensus (RANSAC) to exclude outliers and to calculate the optimal transformation model for the movement between two successive images. The features were clustered into “n” clusters, each of them containing a maximum of 10 well-distributed features and a minimum of 4 features, considering the geographical coordinates. The features in one cluster were selected so that they were not similar to each other considering the Cartesian distance between their descriptors. The features were stored in a Kd-Tree structure to speed up the search time [18]. The matching process is shown in Figure 4.

The projection from the 3D world coordinated on the image plane is shown in Equation (1), where, the projection matrix parameters are the internal parameters calculated in the calibration procedure (focal length *f_x_,f_y_*, central point *c_x_,c_y_*, and the skew parameter α), and the external parameters are calculated from the integrated system (rotation matrix from the camera coordinate to the local level coordinate *R* (*r_11_,…,r_33_*)), and the translation between the point and the camera coordinate *(t_x_,t_y_,t_z_*). Camera calibration was carried out using the chessboard method [19].

(1) $$s\left(\begin{array}{l} x\\ y\\ 1 \end{array}\right)=\left(\begin{array}{ccc} f_{x} & \alpha & c_{x}\\ 0 & f_{y} & c_{y}\\ 0 & 0 & 1 \end{array}\right)\left(\begin{array}{cccc} r_{11} & r_{12} & r_{13} & t_{x}\\ r_{21} & r_{22} & r_{23} & t_{y}\\ r_{31} & r_{32} & r_{33} & t_{z} \end{array}\right)\left(\begin{array}{l} X\\ Y\\ Z\\ 1 \end{array}\right)$$

**Figure 5:**
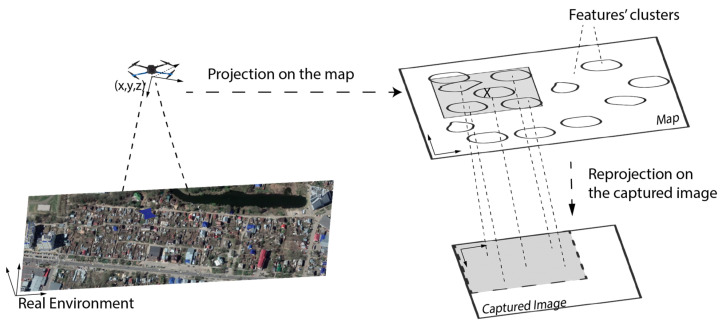
Reducing the matching space, both on the map and the captured image. The searching area on the map was predicted using INS measurements, then this area was projected on the captured image using the camera’s external and internal parameters.

The corresponding 3D world point (X,Y,Z) to its image plane point is known up to a scale “*s*”, which can be solved using an altimeter. The matching took place only between features closer to the reprojected point of the image center on the map with the part of the captured image. Excluding part of the image from feature detection and description is effective since it is a time-consuming process. The cropping and matching procedures are shown and explained in Figure 5. Searching for the matched features that were stored in the Kd-Tree reduced the search time [18].

Remark: In Figure 5, the real environment shown at the left is exactly the place where the experiments took place. It is an urban area where narrow/wide streets exist, along with a lot of houses and trees. During the experiments, the safety factor took the highest priority in path planning because of the low temperatures (<0 °C), wind, and the high load. For more information, refer to Section 6.

## 5. State Dynamics and Navigation Algorithm

The integrated system should satisfy the continuity of the navigation calculations. The INS system represents the process in EKF, while other sensors represent the measurements. The inertial navigation dynamic is presented in Equation (2).

(2) $$\dot{V^{n}}=-([w_{in}^{n}\times ]+2[w_{ie}^{n}\times ])V^{n}+R_{b}^{n}a^{b}+g^{n}$$

where “$g^{n}$” is the gravity acceleration written in the local level coordinates; ”$a^{n}$” is the measured drone acceleration; “$V^{n}$” is the drone velocity expressed at the local level NED ($V^{n}=[\begin{array}{ccc} V^{N} & V^{E} & V^{D} \end{array}]\prime$); “$R_{b}^{n}$” is a 3-by-3 rotation matrix from the body to the local level; [$w_{ie}^{n}\times$] is a skew-symmetric matrix that represents the earth rate expressed at the local level; [$w_{in}^{n}\times$] is a skew-symmetric matrix that represents the rate of the local level coordinates fixed in the center mass of the system (camera coordinate center) expressed at the local level.

Each sensor has its model with derived noise. The barometer measurement model is presented in Equation (3).

(3) $$z_{barometer}=h+v^{bar}$$

where “h” is the height above the ellipsoid, and $v^{bar}$ is white Gaussian noise with zero mean. 

The visual measurement model is also subject to Gaussian noise. The position measurement model is presented in Equation (4).

(4) $$z_{vision}=\left[\begin{array}{l} \varphi_{vision}\\ \lambda_{vision} \end{array}\right]+\left[\begin{array}{l} v^{\varphi }\\ v^{\lambda } \end{array}\right]$$

where “φ,λ” are the geographical latitude and longitude, respectively; $v^{\varphi },v^{\lambda }$ are white Gaussian noise with zero mean. 

The accelerometers and gyros’ biases (*b^a^* and *b^g^*, respectively) are assumed to be random walk and are presented in Equation (5).

(5) $$\left[\begin{array}{l} \dot{b_{3\times 1}^{a}}\\ \dot{b_{3\times 1}^{g}} \end{array}\right]=\left[\begin{array}{l} v_{3\times 1}^{a}\\ v_{3\times 1}^{g} \end{array}\right]$$

where $v^{a},v^{g}$ are white Gaussian noise with zero mean. 

The state space vector contains 12 elements: 3 for position error, 3 for velocity error, and 6 for sensor biases (3 for the three accelerometers, and 3 for the three gyros). The state space vector is presented in Equation (6).

(6) $$x=\left[\begin{array}{cccccccccccc} \delta \varphi & \delta \lambda & \delta h & \delta v_{N} & \delta v_{E} & \delta v_{D} & \delta b_{x}^{acc} & \delta b_{y}^{acc} & \delta b_{z}^{acc} & \delta b_{x}^{gy} & \delta b_{y}^{gy} & \delta b_{z}^{gy} \end{array}\right]$$

**Figure 6:**
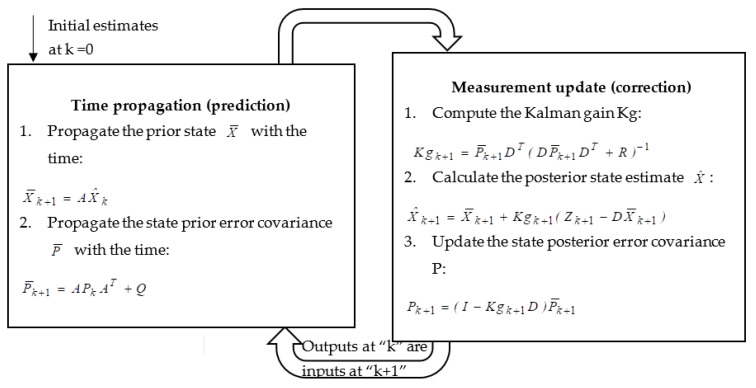
Kalman filter process diagram. Where “*Kg*” is the Kalman gain; “*Q*” is the process noise covariance matrix determined by the inertial sensor’s specification; “*R*” is the measurement noise covariance matrix; “*P*” is the state noise covariance matrix; “*Z*” are the measurements (which should agree with “*D*” as it is sequential filtering) either from the barometer or from the visual system, as shown in Equations (3), (4), and (8).

The discrete state space representation, i.e., the equations of the state and measurements, are presented in Equation (7).

(7) $$\begin{array}{l} x_{k+1}=Ax_{k}+v_{k}\\ z_{k}=Dx_{k}+w_{k} \end{array}$$

where “*A”* is the state matrix; “*D”* is the measurement matrix; *v and w* are white Gaussian noise; “*k”* is the discrete time step. The *D* and *A* matrices are shown in Equations (8) and (9), respectively.

(8) $$D=\{\begin{array}{cc} \left[\begin{array}{ccc} 0_{1\times 2} & 1 & 0_{1\times 9} \end{array}\right] & for\;barometer\;measurements.\\ \left[{\begin{array}{cc} I_{2\times 2} & 0 \end{array}}_{2\times 10}\right] & for\;\mathrm{vision}\;measurements. \end{array}$$

(9) $$A=\left[\begin{array}{cccc} A_{11} & A_{12} & O_{3\times 3} & O_{3\times 3}\\ A_{21} & A_{22} & {\left[R_{b}^{n}\right]}_{3\times 3} & O_{3\times 3}\\ O_{3\times 3} & O_{3\times 3} & I_{3\times 3} & O_{3\times 3}\\ O_{3\times 3} & O_{3\times 3} & O_{3\times 3} & I_{3\times 3} \end{array}\right]\;A_{11}=\left[\begin{array}{ccc} 0 & 0 & -\frac{V^{N}}{{\left(M+h\right)}^{2}}\\ \frac{V^{E}\tan(\varphi )}{\left(N+h)\cos(\varphi \right)} & 0 & -\frac{V^{E}}{{\left(N+h\right)}^{2}\cos(\varphi )}\\ 0 & 0 & 0 \end{array}\right]\;\;\;\;\;\;\;\;\;A_{12}=\left[\begin{array}{ccc} \frac{1}{M+h} & 0 & 0\\ 0 & \frac{1}{\left(N+h)\cos(\varphi \right)} & 0\\ 0 & 0 & -1 \end{array}\right]\;A_{21}=\left[\begin{array}{ccc} -2w^{e}\cos(\varphi )V^{E}-\frac{{\left(V^{E}\right)}^{2}}{\left(N+h)\cos^{2}(\varphi \right)} & 0 & -\frac{V^{D}V^{N}}{{\left(M+h\right)}^{2}}+\frac{{\left(V^{E}\right)}^{2}\tan(\varphi )}{{\left(N+h\right)}^{2}}\\ 2w^{e}(\cos(\varphi )V^{N}-\sin(\varphi )V^{D})+\frac{V^{E}V^{N}}{\left(N+h)\cos^{2}(\varphi \right)} & 0 & -\frac{V^{D}V^{E}+(V^{N}V^{E})\tan(\varphi )}{{\left(N+h\right)}^{2}}\\ 2w^{e}\sin(\varphi )V^{E} & 0 & \frac{{\left(V^{N}\right)}^{2}}{{\left(M+h\right)}^{2}}+\frac{{\left(V^{E}\right)}^{2}}{{\left(N+h\right)}^{2}}-\frac{2g_{0}}{r+h} \end{array}\right]\;A_{22}=\left[\begin{array}{ccc} \frac{V^{D}}{M+h} & -2w^{e}\sin(\varphi )-\frac{V^{E}\tan(\varphi )}{\left(N+h\right)} & \frac{V^{N}}{M+h}\\ 2w^{e}\sin(\varphi )+\frac{V^{E}\tan(\varphi )}{N+h} & \frac{V^{D}+V^{N}\tan(\varphi )}{N+h} & 2w^{e}\cos(\varphi )+\frac{V^{E}}{N+h}\\ -\frac{2V^{N}}{M+h} & -2w^{e}\cos(\varphi )-\frac{2V^{E}}{N+h} & 0 \end{array}\right]$$

where *I_3x3_* is the identity matrix of the 3-by-3 dimensions; *O_nxn_* is the zero matrix of the n-by-n dimensions; *M* and *N* are the radiuses of the curvature of the Earth in the meridian and prime vertical directions, respectively; “*w*^e^ “ is the earth rate; “*r*” is the mean radius of the earth. A diagram of the KF process is shown in Figure 6. The full derivation of the state space equation of KF is found in [3,20].

The measurement model has two parts—the barometer, which works at 50 Hz, and the visual system, which works at approximately 24 Hz, while the process model is represented by the IMU, which works at 100 Hz. The integrated navigation pseudocode is shown in Algorithm 1.
**Algorithm 1.** The integrated navigation algorithm**Inputs**: Initial position from GPS, Euler angles from magnetometers, barometer and inertial sensors’ outputs, images, and the map. **Outputs**: Position and velocity at local-level coordinates. **Pseudo code**:1. **Initial Alignment** 2. **while** (Vision is not available)      Propagate the system state over the time using INS(). 3.       **If** (barometer is available)            EKF_ update_ height(). 4.       **If** (Image is available)           t_c_ = time ()           Project the INS position on the map clusters (INS, map).           Project clusters’ centers on the captured image (image, map clusters, INS).            Features detection and description (SURF, cropped part of the image).           Features matching using (map clusters, cropped image).           Visual navigation (PnP, RANSAC, matched features). 5.       **If** (vision solution is ready)            t_n_= time()            Go to 6. 6. **If** (vision solution is ready)            EKF_ update (INS, Computer vision, t_c_)            EKF_recalculation from t_c_ to t_n_().            Compensate INS drift().            Compensate for the accelerometer bias().            Compensate for the gyro bias(). 7. **Return to 2.**

The inertial navigation process starts with initial alignment. The GPS position is used to calculate the initial position. The magnetometer is used in the alignment procedure to calculate the transformation between the IMU and the local coordinate system, and then INS navigation starts. When an image is available, the INS solution is projected onto the map, and a matching process starts between the suitable clusters on the map and the detected features in the targeted part of the captured image. When the visual results are ready, the EKF recalculation is performed from the capturing “t_c_“ moment until the current moment “t_n_”. The height channel of the INS system is compensated by the barometer measurements. The recalculated sequential KF is referred to as RSKF.

## 6. Implementation and Realization

**Figure 7:**
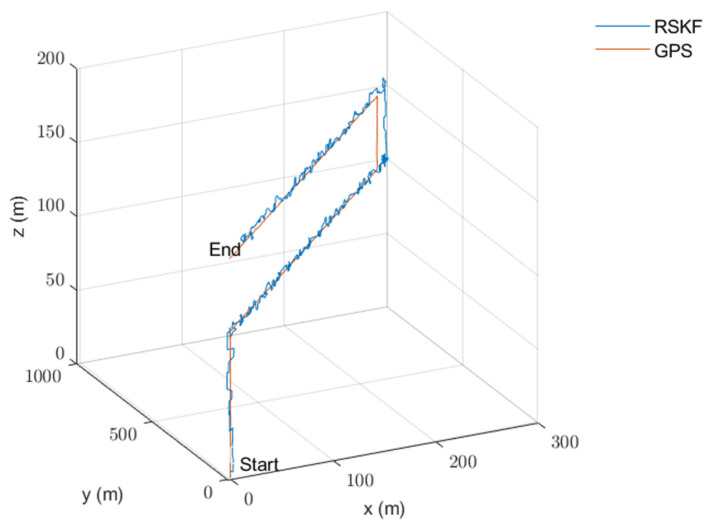
The calculated integrated navigation 3D path using RSKF with the reference 3D path.

**Figure 8:**
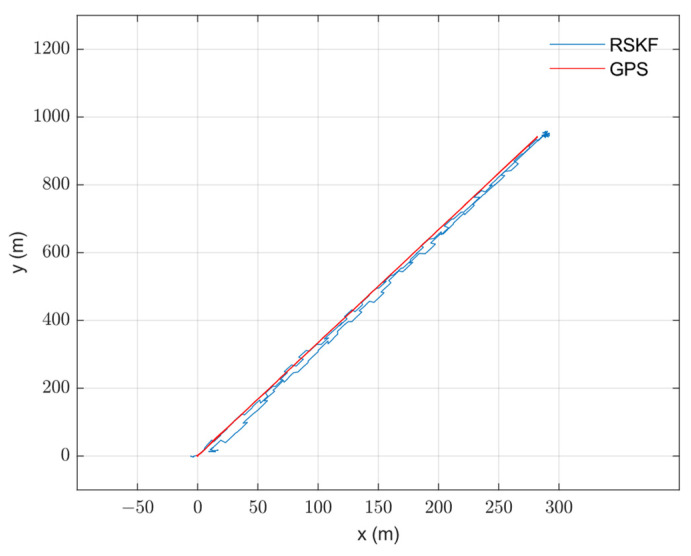
The calculated integrated navigation horizontal path using RSKF with the reference horizontal path. The closed path in the horizontal plane is a good indicator of the INS drift compensation.

**Figure 9:**
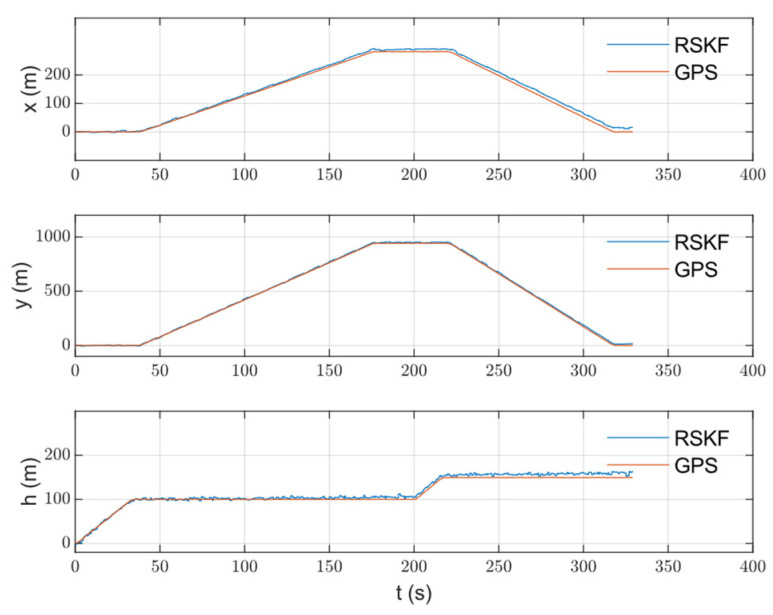
The calculated RSKF position with the reference position on the three axes.

**Figure 10:**
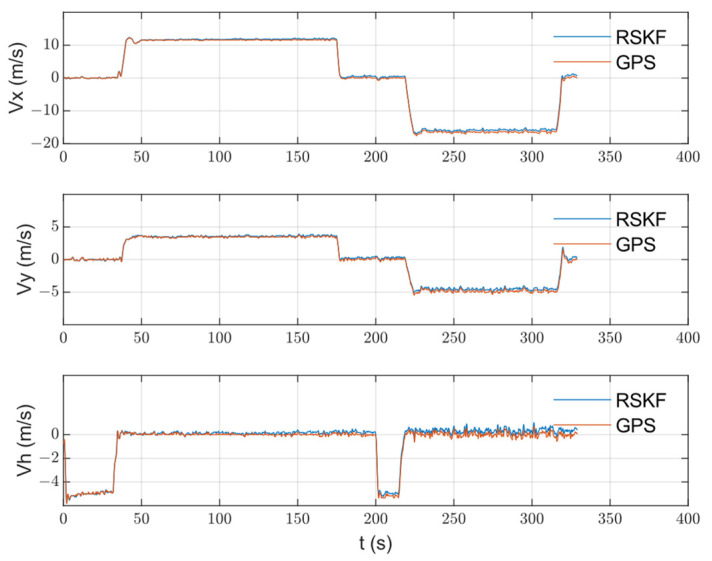
The calculated RSKF velocity with the reference velocity on the three axes.

**Figure 11:**
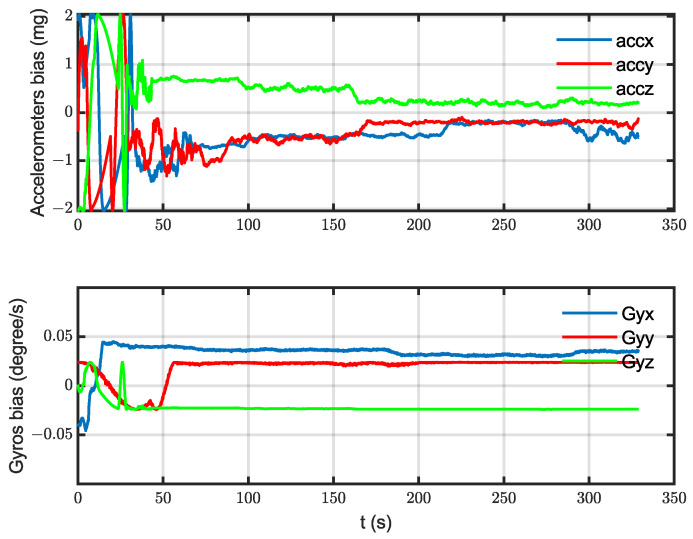
The estimated bias for the 6 sensors: 3 for the gyros and 3 for the accelerometers.

The algorithm was tested on data collected from an urban area using a DJI Matrice 600 Pro drone. The drone was equipped with an IDS camera with a 6 mm focal length lens, Xsens MTI-710 g IMU (which has a 100 Hz output rate), a barometer and compass (included in the IMU box), and an intel NUC computer. The GPS measurements were used as a reference. OpenCV was used for image processing under Linux. The image and IMU data were synchronized using time stamping. The reference map was prepared from Google Maps. The drone flew on a path of about 3 km in length, with an average speed of 12 m/s, and an altitude between 100 and 150 m for 5.5 min. After that, the data were processed, and the 3D path, 2D path, position, and velocity of the drone were calculated and are shown with the reference measurements in Figure 7, Figure 8, Figure 9 and Figure 10, respectively. The sensors’ biases are shown in Figure 11. The NED axes are referred to as x, y, and z, respectively, in meters.

During the climbing stage, only measurements from the barometer were used in the integrated navigation system. The visual navigation system was not working until the drone reached the nominal altitude, i.e., until 40 s. As a result, a weak estimation of the sensors’ biases was expected. In Figure 11, a transient state is obvious at that stage. When the visual measurements were available, a good estimation of the errors started, as shown in the same figure. The same consideration was applied to the velocity and horizontal position because error compensation at the climbing stage was carried out only on the vertical channel (or the height).

## 7. Results Analysis

The integrated navigation system position results were accepted with an RMS position error of less than 3.5 m. Since the vision measurements were not available until the drone finished climbing to the nominal altitude (100 m), an error growth with a transient state was obvious in the climbing stage (until ~40 s). That transient state can be seen in the velocity, position, and bias estimations. Furthermore, the weak maneuvering over the path, especially during the climbing stage, affected the filter’s prediction ability. In the steady state, the sequential filtering worked well in compensating the INS drift with small vibrations that occurred when updating the state with the barometer and vision measurements. The vibrations were expected during the error-correcting phase, and they could be avoided by averaging filters, but they were accepted in this work. Although the velocity had no direct update (measurements), compensating for the sensor’s errors resulted in accurate velocity measurements. The sensor errors were approximated by random walk “bias” derived from white Gaussian noise. Of course, the real error models were more complex and contained other errors, such as scale errors, and they were not necessarily derived from white Gaussian noise. The experiments for the data collection were performed without any laboratory calibration for the IMU, and in rough weather conditions, when the temperature was about 0˚c. All the aforementioned factors affected the system and sensor characteristics, but the resulting values of the bias estimation practically gave measurements that improved the position and velocity accuracy.

The image-processing and visual navigation calculations consumed an average time of 42 ms using the SURF features in the mutual aiding mode, compared to 65 ms with the normal working mode, i.e., it was about 35% more efficient on the same CPU. The time improvement was the direct result of the map offline processing and the mutual aiding, which reduced the search space on both the reference map and the captured image. Filter recalculation, by updating the state with the visual measurements at the capturing moment and recalculating the KF to the current moments, solved the error that resulted from delayed updating, i.e., the case where updating was carried out when the measurement was ready after processing. The recalculation process required storing the measurements for the processing period, which was not a heavy load for 42 ms, considering the required memory. Sequential filtering has good flexibility to update the state with different sensor measurements as they arrived. It also simplifies the calculation by turning them into a lower-dimension matrix. One outcome of sequential filtering is that sequentially updating the state from different sensors with different characteristics might lead to some vibration or noise, which could be avoided by averaging or could simply be accepted according to the application [21].

## 8. Conclusions

This work presents the implementation of an integrated navigation system based on inertial sensors, a vision system, a barometer, and a compass. The mutual aiding mode was adopted. While the vision system was used to correct the inertial system drift and compensate for the inertial sensors bias, the INS was used to enhance the matching process in the vision system by bounding the searching area in the image-matching process. The reference image was processed offline, and SURF features were clustered and stored in a Kd-Tree structure to speed up the search time. The system’s delays were analyzed, and it was suggested that image-processing time-delay errors were solved using the recalculation technique from the image-capturing moment. Sequential EKF was used with recalculation for a period that was equal to the image-processing time. The system was implemented using data collected from a real flight in an urban area using the DJI Matrice Pro 600 drone. Because of the difficult weather conditions during the data collection (temperature < 0 °C and wind), high priority was given to the safety factors in path planning over the urban area. The final results show the ability of the system to maintain the continuity and the accuracy of the navigation solution without using GPS and with a position error of less than 3.5 m RMS. The feature clustering of the map with mutual aiding reduced the matching time for the visual navigation system. The sequential filtering helped to incorporate all sensors in state updates at any time they arrived. The recalculation of the EKF solved the errors resulting from the image-processing time delay and enhanced the accuracy of the overall navigation system. The recalculation was not time-consuming since a large number of the recalculation operations were carried out using the barometer measurement, i.e., scalar EKF, and only one visual recalculation in each recalculation step was required.

The adopted algorithm succeeded in enhancing the image matching (by INS projection on the map and cropping the useful part of the captured images). Furthermore, it was effective at solving the image-processing delay problem using the EKF recalculation. Visual inertial odometers are effective in maintaining the continuity of measurements when they are integrated with GPS, especially in tight coupling integration, as shown in the work presented in [22], where they obtained good results and maintained high continuity. However, GPS is subject to a provider even in the tight coupling mode. In addition, odometers (inertial and visual) suffer from incremental errors that need to be compensated for using an absolute navigation system, such as GPS or scene matching. Compared to odometer-based methods, integrating INS with scene matching in this study gave good results after enhancing the processing time and overcoming the processing time delay by filter recalculation. Since scene matching is not subject to a provider, the overall system (mutual-aided INS/scene matching) has much more independence than the case of including any GNSS in the navigation system. 

Although filter recalculation was proven to be able to maintain accuracy even with variant delays, it has the problem of computation burden compared to other delayed fusion methods, such as the Larsen method [10]. Larsen-based methods require calculating a correction gain, and they have almost the same complexity that EKF has. In this work, depending on the sequential filtering, which reduces the matrix dimensions, and having only a barometer measurement in addition to the visual measurements (which are a matter of interest) encouraged the use of the simple recalculation principle, since it is easy to measure the required capturing and measurement-ready moments. The suggested method of recalculation proved its efficiency in delay error compensation.

The implemented RSKF-based system could be used in areas where GPS outages are expected for navigation purposes in surveillance, path-tracking, rescuing missions, and mapping when the carrier position is included in the process. The suggested method for employment of the image information in its real accurate time and accurate carrier position could also be applied to produce accurate maps and DSMs easily and to make the automated or intelligent search and rescue missions much safer and more powerful. Future works will focus on realizing online experiments, where it will be more efficient to test the recalculation method online in the real world.

## Data Availability

Not applicable.
